# Competitive Debate: A Successful Inter-team Gamification Experience in the Human Resources Subject

**DOI:** 10.3389/fpsyg.2021.708677

**Published:** 2021-12-10

**Authors:** Guillermo A. Sánchez Prieto, María José Martín Rodrigo, Antonio Rua Vieites

**Affiliations:** Faculty of Economics and Business Administration, Universidad Pontificia Comillas, Madrid, Spain

**Keywords:** communication skills, human resources, gamification, presentations, teaching innovation, competitive debate

## Abstract

Students demand more active and participating teaching innovation methods, and activities such as presentations are not enough to satisfy those demands. In this research, competitive debate is used as inter-team gamification with third year students from a Business School studying the Human Resources Management subject. Out of this experience, qualitative and quantitative data are obtained. Results reinforce the continuation of classroom competitive debate due to the evidence of its motivational, learning, and communication skills improvement, and knowledge acquisition effects. The possibility of application with actual professionals is seriously considered.

## Introduction

New student generations, as well as workers, demand teaching and training in more dynamic and participative ways. The millennial thinking, linked to active participation and decision-making capacity, influences this way of understanding work and education. Thus, this generation, in its intuitive learning model, looks for almost immediate results and compensation which provokes the use of gameful resources and active results as one of the more effective learning methods for these new generations (Rodríguez-Casado and Rebolledo-Gámez, 2017). For this generation, learning through videogames is one of the principal learning strategies together with mobile learning and gamification. In relation to this last method, gamification, there is not a clear consensus in the scientific community about its definition and usability in learning environments (Deterding et al., 2011; Seaborn and Fels, 2015) although Kapp (2012b) defines it as “the use of mechanics, aesthetics, and thinking of games to engage, motivate, promote learning, and resolve problems.” Seaborn and Fels (2015) conclude that “gamification has two key ingredients: it is used for non-entertainment purposes: it draws inspiration from games, particularly the elements that make up games without engendering a fully-fledged game” in this line, inter-team competitive gamification through competitive debate is used in this experience to teach and grade the knowledge about training in human resources (HR), not just to play, which is what debate tournaments are after fundamentally. To Dichev and Dicheva (2017), gamification is “the introduction of game design elements and gameful experiences in the design of learning processes” which is exactly what was done in this case, taking the activity of competitive debates and applying it to a classroom to learn some specific knowledge. After reviewing numerous papers, Huotari and Hamari conclude that gamification refers to “a process of enhancing a service with affordances for gameful experiences in order to support users' overall value creation” (Huotari and Hamari, 2017). That is the idea for debate in the classroom, which should deliver a gameful experience with which students find an added value for their learning. Based on another four works, Zainuddin et al. (2020) define gamification as “the process of applying game elements to non-game contexts” and in this case it is taken as a game, a competitive debate, with most of its elements and is being applied to the classroom context for learning and grading. From a concept point of view and in the specific case of competitive debate applied to education, it fits into the category of gamification according to Sánchez et al. (2020). In this case, inter-team gamification refers to “groups of players compet[ing] with other groups and thus several players share the goal to jointly obstruct[ing] the goals and actions of others” (Morschheuser et al., 2019). That is exactly what happens within a debate, one team explains reasons pro or affirmative and the other team presents the con or negative reasons of a subject during the first shift, later on, each team has to perform rebuttals against each other. At the end, a jury appoints a winning team.

Likewise, numerous studies endorse the positive relation of debate and critical thinking. Some of the studies have proved this relation in different countries and different educational environments (Allen et al., 1997; Darby, 2007; Mubaraq, 2016; Celada-Perandones et al., 2018). Because of that, debates are a useful tool in the development of critical thinking since an individual who thinks critically tends to live rationally, reasonably, and empathetically (Núñez-López et al., 2017, quoting Willson, 2012). However, some authors such as Greenstreet (1993) considered, with evidence, that the relationship between debate and critical thinking was not sustained.

Up-to-the-moment questions, such as if debate is a valid communication skills training technique or valid to teach certain knowledge, have been researched on the educational environment especially in higher education (Reverter, 2018). As far as our bibliographic search shows, the only experience of debate with professionals was conducted by a group trained in communication skills from the State Department of the United States of America (Benton, 2015). These results only measured participants learning sensation.

After reviewing literature about gamification, several demands are observed. The scientific community demands changes and reorientations in the study of gamification. Some authors ask for new gamification tools that are more personalized and smaller in size (Ortega-Arranz et al., 2017). In that sense, a competitive debate is always unique and unrepeatable. Contrary to the fact that in certain video games in which the player always goes through the same levels or in a test with points where players find the same questions repeated, each debate is unique, due to the interaction, competition, and collaboration. Others claim to investigate the application of dynamics that require low technology (Rapp et al., 2019; Zainuddin et al., 2020). This is exactly the case of the competitive debate, in which computer technology is not necessary, so this demand can be tested with competitive debate. It has been proposed, although not in a massive way, to investigate the user experience when testing gamification (Klock et al., 2020) and that is what is done in this study. Likewise, the need to carry out empirical studies is expressed in order to conduct research with more accuracy (de Sousa Borges et al., 2014; Dichev and Dicheva, 2017). Although it is true that in this case we are just presenting a first experience, the next step of this work would be to test the degree of effectiveness of competitive debate, for example, when training professionals in communication skills. It would be done through an experiment, with a control group and several experimental groups. Through our literature search, we found that the greatest demand is to deepen the human interaction in gamification, as well as work in collaborative-competitive environments (Burguillo, 2010; Huotari and Hamari, 2011; Rapp et al., 2019; Sailer and Homner, 2019). It is precisely in competitive debate that one competes against the other team, and it is necessary to collaborate within your own team. In addition, human interaction, through dialectical exchange, is the cornerstone of it. All of this is a clear example of human interaction in competitive environments. Besides, inter-team gamification has been “largely ignored by gamification” (Morschheuser et al., 2019) and needs to be researched (An, 2020).

From the literature review, it can be stated that there is research about gamification, competitive debate, and training in communication skills but they exist independently from one another. This paper intends to combine these three elements, obtaining and applying a model by observing and analyzing different aspects related with the experience of students and their perception about this kind of teaching practice related to learning acquisition. In addition, through literature searches we determined that publications on gamification are mainly related to IT, science, and engineering, although the domain of computer science is clear (Dichev and Dicheva, 2017; Swacha, 2021). However, there are hardly any publications on experiences in social science University subjects such as the experience reported in the area of HR in this case.

The objectives of this research are:

a. Determine if competitive debate inter-team gamification improves the learning sensation of the contents by students, more specifically in the HR subject.
b. Determine if using competitive inter-team gamification through debate, students have the sensation of improving their communication skills.
c. Predict the potential receptivity of inter-team gamification through competitive debate as the training method of inter-team gamification for future business managers.
d. Analyze the receptivity toward competitive debate by students as a grading method compared with other ways (papers, exams, presentations, or group papers).

Gamification is becoming a trend in the HR training environment (Osipov et al., 2007; Kapp, 2012a; Ramírez, 2014). Likewise, such applications are universal in the environment of marketing, sales, productivity, and workers motivation. Some of the most common elements of gamification are usually points, rewards, leaderboards, etc. but this case has focused on applying the competitive part of inter-team gamification through debate and has not emphasized more common elements. Attention has been paid to elements that are not often researched such as competition, collaboration, the use of low technology, and inter-team gamification. As a matter of fact, looking for inter-team gamification in Google offers just 151 results. On the other hand, it was the intention of the research team to not focus on the way in which gamification is implemented but on the essence of gamification, the use of games in non-gaming contexts (Kapp, 2012b).

In reference to gamification and communication skills, the studies that have been found in this research mainly focus on virtual reality and gamification techniques focused on reducing anxiety or fear of public speaking rather than teaching communication. It is more therapeutic than formative.

In reference to debate applied in a teaching environment, experiences have been performed in higher and not higher education. Several studies have been conducted about classroom debate in numerous academic disciplines. Specialties such as engineering, law, or nursing have used debate as a teaching tool, although not as a grading tool (Mitchell, 1998; Bellon, 2000; Kishida, 2003; Abhijit and Macchiette, 2005; Merrell et al., 2017; Sapitri, 2017; Galiano et al., 2018).

Only one reference proposes a competitive classroom debate model as a tool in the HR subject (Sánchez, 2017). However, such a model does not present empirical references or experiences about its application. The present work presents some necessary evidence to discuss the feasibility of competitive debate at a University classroom for a HR subject.

Benton's experience (Benton, 2015) was the only available use of debate for professional training purposes found in our search. In his research, performed at the State Department of the United States of America, debate was used as a training tool for communication skills with department workers.

## Participants and Steps to Perform a Competitive Debate in the Classroom

Forty-two students studying HR in their third year of the Business Administration degree were chosen for this experience. The group was balanced in terms of gender. When the experience took place, March 2019, the teams were already prepared as they had had to perform presentations about different training solutions in their HR development course since the beginning of the semester. Each team was formed of four or five students, and each team was asked to research and present a new training trend in business such as:

- Corporate Universities
- Personalized Learning Environments
- E-learning
- Massive Online Open Courses
- Mentoring
- Coaching
- Outdoor training
- Gamification.

Thus, each team, after a research and preparation period, was asked to defend their training solution and attack the one of the opposing team with the purpose of persuading a jury about which tool was more powerful as a training method. The matches were made in a way that training solutions belonged to categories as alike as possible. Thus, matches were as follows: Corporate Universities vs. Personalized Learning Environments (PLE), E-learning vs. Massive Online Open Courses (MOOC), Mentoring vs. Coaching, and Outdoor Training vs. Gamification.

Students received 2-h basic debate training. In this training, the professor of the subject was present but did not have an active role. The other trainer, specialized in debate, was introduced to the class as a consultant not as a scientist. This trainer explained fundamental concepts related to the debate format: opening presentations, rebuttals, counter-rebuttals, and conclusions. Likewise, the difference among debates about facts, values, or solutions, which are the three types of debate issues according to their nature, was explained (Cirlin, 1999). The training had a practical part in which steps to develop a debate classroom for the HR subject explained by Sánchez (2017) were executed by students. Table 1 explains the previous training structure before the debates took place. In this training, it was explained how points were achieved and how these points could lead to a better grade as a reward.

**Table 1:** Debate training timing.

**Issue**	**Explanation**	**Time (min)**
Introductions	What is the goal of the activity and what is it about. Professors introductions.	10
Debate issue or resolution	The goal is proving the jury what training solution is better. Debate is presented as training and grading tool.	10
Format, times, and mechanics of the debates	What is each debate part for and how much time lasts. •4 min. Opening presentations, •5 min. 1st refutation, •5 min. 2nd refutations •3 min. Conclusions	10
Research	Explaining the evidence concept and practice of evidence research.	15
Argumentative lines design	Debate teams must outline their three or four ideas about why their training solution is better than the other solution.	15
Debate rubric explanation	Explanation of rubric with Q and A.	15
Disposition of argumentative order almost definitive	Teams define openings, argumentation development and conclusions for their speeches.	15
Positions assignment	Each team decides what team member does what during the debate and in what order.	5
Organization of tasks among students	Each team decides who does what during the week before debates on research and others tasks.	10

*Source: self-elaboration*.

One week before the debates, students prepared their debates by researching about their training trend, looking for strengths of their training solution and weaknesses of the opponent's solution. During this week, students' work was not supervised. Students were free to work autonomously as would be the case for a written paper or a classroom presentation.

Each debate had two teams while two other teams acted as the jury. Finally, whether a team won was be decided by a hand raising vote by the teams. The jury's opinion was not considered because it would have slowed down the development of the debates due to the brief time available for reaching a decision. The professor of the subject used the judge's rubric to grade the teams, so winning meant a reward on the grade.

## Research Methodology

The strategy was to quantitatively and qualitatively measure students' perception of the experience. A mixed methodology was implemented, in which quantitative and qualitative tools were used. This methodology combined numerical and verbal or textual data in order to understand complex problems. For Hernández Sampieri, learning is one of those problems that justifies a mixed approach, as it provides more value than a single approach and accurate information. In this case, we faced a situation in which an attempt was made to explain something as subjective and experiential such as a game experience with inter-team gamification through competitive debate applied to HR learning (Hernández Sampieri 2016, pp. 534–536). Similar experiences in which the opinion of students was measured with a mixed method regarding gamification experiences can be found in previous research (Lopes et al., 2019; Ndlovu and Mhlongo, 2020).

As for the sample, 42 students from the HR class of the Business Administration degree participated in the experience. Regarding the gender of the participants, this was balanced. The average age of the students was around 21 years. This sample included the participants of the experience, although the number of responses to the questionnaires was less, as will be seen. The sample is superior to other studies on gamification in the University environment such as de Almeida Souza et al. (2017) in which a sample of 18 students was used, from whom quantitative results were obtained and within these, 6 collaborated in in-depth interviews. In a study by Ndlovu and Mhlongo (2020), 31 responses from 34 students were obtained when measuring satisfaction. The case of Scholz et al. (2021) used gamification in a history course in the third year of University and provided a sample of 15 participants of which they obtained results from 12 respondents, also using a mixed method.

A descriptive analysis of the measured variables (percentages, averages, standard deviation) and parametric techniques (Student's *t*) or not (Chi square), according to circumstances, were used to determine if responses presented a bias different to indifference. Thus, for instance, if the item was measured from 1 to 10 it was considered a parametric contrast if the average was 5.5 in the *t*-test for one sample. Thus, hypothesis rejection implies that students' opinions would lean significantly toward mean values over the average or below the average, but not remain as an average group indifference when answering a question. In the case of ordinal variables with a lower number of categories, Chi square was used that shows a null hypothesis of the percentage distribution of the categories as uniform. So, if the hypothesis was rejected, the group was sensible to the question or opinion asked. Also, it was analyzed if there were differences marked by dependent variables, with a contrast test for equality of means or the Mann-Whitney test.

It is necessary to take into account that there are almost no methodological references due to the lack of publications that combine the mentioned elements (inter-team gamification, low technology gamification, debate, and HR teaching at University).

A set of items was defined as well as a set of other elements.

Questions to evaluate or measure:

- Intention of attendance to HR subject after the experience
- Interest for training as part of the HR subject content
- Interest for the specific training solution they defended in the debates
- Interest or curiosity by debate and personal communication
- Sensation of capacity to perform public speaking after the experience
- How entertaining was the activity for students
- Comparison of the debate activity as a grading tool to: exams, group papers, individual papers, presentations in class
- Recommendation or not of the activity to other students
- Relevance of the activity in a real company to teach communication skills, decision-making in a team, or talent detection.

Elements that may condition perception variables that can condition the other variables:

- Previous participation in competitive debate
- Role or function performed in the debates (starting presentation, conclusion, jury…)
- Natural proclivity for public speaking
- Gender
- Approximate average grade
- Nationality
- Professional goals.

The research team ruled out hypotheses due to the limited literature on the three elements together: competitive debate, HR teaching, and inter-team gamification. Likewise, the aim of the article was not to measure skills or look for causalities, the objective was to measure receptivity using a gamified technique through inter-team competition with the allocation of points and prizes. Regarding data processing, SPSS version 24 was used.

Regarding the qualitative part, an open evaluation form of the activity was distributed through Moodle after the whole experience was completed. The evaluation asked for testimonials about what they liked from the activity and what did not, through a personal, free, and voluntary written reflection. They were not asked to evaluate or consider any specific aspect of the experience.

The development of the debates went on without any remarkable incidents. Just to mention, some of the exchange students were not aware of certain aspects of the debate format. For instance, two foreign students understood they could share the floor in the same shift, something not allowed by the rules of these debates. A certain passiveness was observed in some students when they were not debating, this meant they were watching the debates as part of the public.

**Figure 1:**
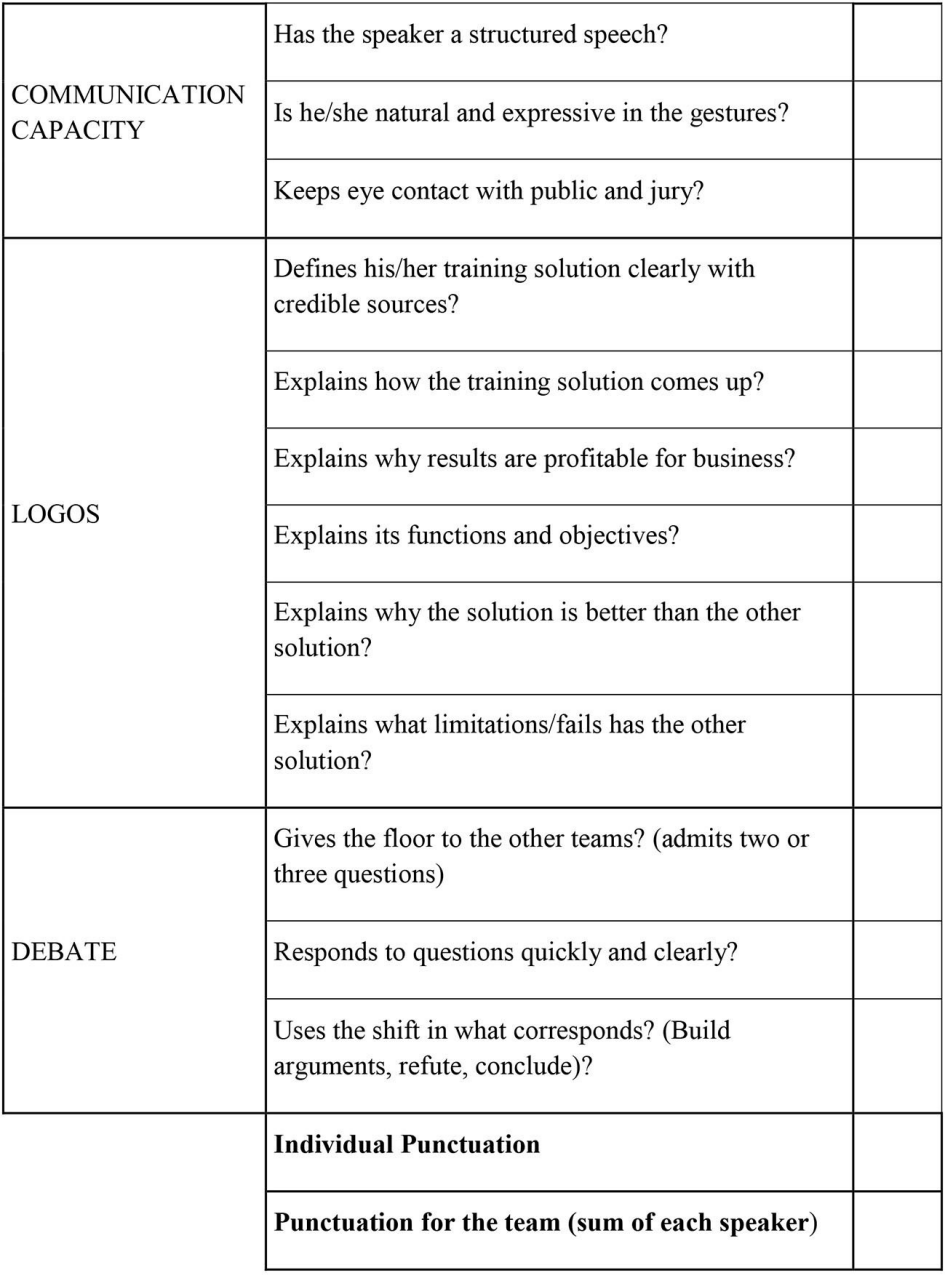
Rubric of evaluation for debates. Source: Self elaboration.

A grading rubric was used, as explained in Figure 1, so the professor and students who acted as a jury, could adequately evaluate the debates; this constituted the point allocation part of gamification. The rubric measured three competences: communication capacity, logos, and debate capacity. The scale to measure each item was zero (unsatisfactory) or one (satisfactory), this way the professor and judges could grade faster than if they had to grade each item from 1 to 10. Thus, in order to grade each student, the professor added the points that each student obtained to what a student would get as a grade. For instance, if a student accumulated 12 points, he would get a 10, the maximum grade in Spain, and a student that obtained 5 points would get a grade of 4. The professor could also grade according to the team score. Thus, if the team accumulated a lot of points by complying with many items, the grade would be higher.

## Analysis, Results, and Discussion

The quantification of results was conceived by measuring students' opinions about the technique used in the classroom with an evaluation questionnaire. The qualitative part was completed with students' written testimonials without any specific guidelines. Following this, quantitative results were taken together with the qualitative results. Finally, we were unable to obtain the evaluation of all participating students in the debates because some missed class the day in which the questionnaire was handed out so finally, out of 42 participants, 33 responses were obtained which constitutes a certain limitation and does not allow us to generalize. Following this, the quantitative and qualitative responses given by students were analyzed.

### Intention of Attending the Subject

Students were asked about their intention of attending the subject after the debate activity. This question was measured with an ordinal scale: 1 more than before, 2 same as before, 3 less than before. It was intended to test the hypothesis that participating in debates stimulates attendance to class. However, this question may hide the sociological principle known as “socially desirable response bias.” This means, the students may respond in a way that is appealing to professors, researchers, or give any response that may be considered socially desirable. Thus, if we consider the response as valid, the debate activity motivates students to attend more than before in 36% of students, same as before in 61% of students, and less than before in 3% of students. It was revealed that a predisposition to attend class more than before (*t* = −3.546 *p*-value = 0.001; Chi-square = 16.545, *p*-value = 0.000) existed in the students. Table 2 shows the statistical summaries for each one of the following variables. It corroborated one of Monarca's affirmations (Monarca, 2013) when explains that classroom participation improves the teaching proposal that it generates. The conclusions of other gamification papers, including computational gamification, in which participants showed more interest after they gained the experience of gamification are confirmed, although in this case, we just determined the intention of attending the subject (Laskowski and Badurowicz, 2014; Lister, 2015; Pinter et al., 2020).

**Table 2:** Interest in training, their training solution, and debate/communication.

	**Training**	**Issue**	**Debate**	**Entertainment**	**Communication improvement**
**Scale from 1–10**	**Percentage**	**Percentage**	**Percentage**	**Percentage**	**Percentage**
1	6.1	3	0	0	15.2
2	3	0	6.1	3	0
3	6.1	6.1	6.1	6.1	15.2
4	3	6.1	6.1	0	9.1
5	18.2	30.3	21.2	12.1	18.2
6	30.3	18.2	12.1	15.2	12.1
7	21.2	15.2	24.2	18.2	15.2
8	12.1	15.2	9.1	21.2	12.1
9	0	3	9.1	15.2	3
10	0	3	6.1	9.1	0
Average	5.61	5.91	6.18	7.00	4.91
*p*-Value (Ho mean 5.5)	0.0747	0.216	0.079	0	0.15
Standard deviation	1.870	1.860	2.100	2.031	2.363
*N*	33	33	33	33	33

*Activity entertainment for students and improvement on communication. Source: self-elaboration*.

### Interest in Training, Training Solutions, and Communication

In this part, we analyzed the intensity of students' interest in three aspects related to the debate: interest in training as part of the subject content, interest in the training solution they defended (*e-learning*, corporate universities…), and interest in debate and communication *per se*. The 1–10 scale measured the degree of interest for each item (training, training solution defended, and debating and communication) from 1, the interest did not increase at all to 10, my interest increased a lot more.

Although a clear interest increase was apparent in the three mentioned aspects, since averages are around 6, these averages are not very high or significantly superior to the 5.5 value. Thus, the testimonial of a student may help to discover why interest may be lost when students are in the classroom watching debates as spectators: “*As learning material to me it seems bad. Maybe, the presentation that you have that to do and the issue you are going to speak about you have to master it, besides the disadvantages that may have the competition. That really does help for learning. The problem comes when you have to pay attention to other debates. In my particular case, out of the other three debates performed in class, one I did not pay attention (I was thinking about something else), and in the other two I was more attentive to how speakers spoke than what they said in order to discredit the rest, than learning as I would learn in a classroom about an issue*” (C1).

In general, in the three measured aspects, it was observed that superior punctuation, from 6 to 10, accumulated more than half of the responses. To a greater extent was the interest on training (63%), while the interest in the training solution they defended was 54% and the interest on debate accumulated 60% of the responses superior to 6.

On the other hand, another student spoke about the effort of joining the experience, although they perceived learning benefits and showed motivation “*The experience was very good since I love to do new things and learning, above all to prepare a debate, the search for evidence and the preparation facing possible arguments of the opponent, requires of a good strategy and that I love. On the other hand, the research work behind is very long and it was performed in a week with mid semester exams of all the subjects and with other papers to turn in and that was very complicated to manage and organize*” (C2). This finding aligns with the results obtained by other researchers such as Dabrowski et al. (2015), Lopes et al. (2019) and Ndlovu and Mhlongo (2020) in which students declared a higher interest of the content of the subject.

All the engagement has an explanation based on gamification mechanics. The reasons why competitive classroom debate provoked student engagement is that competitive debate complies with most of the 10 mechanics that Werbach and Hunter explain as causing this engagement (Werbach and Hunter 2012, p. 79). Challenge (the task is clear and is a challenge, to convince the jury who is right). There is a chance (not so much in terms of the appearance of random elements from the game itself, but in terms of what the other team can do which is unpredictable). Competition (one team wins and other loses the debate). Cooperation (team members must cooperate with each other to achieve victory). Feedback (from the jury, the team members, their classmates, and the teacher). Acquisition of collectible or useful resources (they are not present if we consider them as elements that are obtained in the game and that help to achieve the goal). Rewards (the grade may be better depending on how they perform in the debate). Transactions (as such there are no transactions, in terms of elements of the game that can be exchanged, but there is interaction between both teams). Turns (initial exposure, rebuttals, etc.). Win states (only one team will have the vote of the jury). This compliance with most of the mechanics explains the engagement of the students, if we consider that mechanics are those basic processes that drive action and generate player engagement (Werbach and Hunter 2012, p. 80).

### Perception of Capacity Improvement for Public Speaking

In this part, we measured the sensation of learning in respect to public speaking. Thus, students were asked to what degree they considered they improved their capacity for public speaking in a scale from 1 (same as before) to 10 (a lot more). Most of them thought they increased their capacity for public speaking; only 15% of the cases thought they were just like before in respect to their communicative capacity. Thus, the percentages of higher punctuation, among 6 and 9, constitutes a bit >42%, while percentages ≤ 5 are something >57%. These data confirm that students perceive a clear advance in their public speaking capacities, superior to 5.5 (*p*-value = 0.161), maintaining the opinion at a medium point, mean of 4.91. If compared with other papers of similar populations, we will find different results. Thus, in the case of a digital app, 37% of the students found the app useful for presentations when learning English (Barrett et al., 2021). The percentages about perceived usefulness obtained in our study about gamification and HR are superior.

### Entertainment of the Debate Activity

One of the considerations that the research team took into account before the experience was that competitive debate in the classroom could be an entertaining activity. Students were asked up to what point this activity was entertaining for them, on a scale from 1 (not entertaining) to 10 (very entertaining). Thus, more than 90% of the sample responses were above 5, confirming it as a clearly entertaining activity, notably overcoming the uniform mean value of 5.5 (*p*-value = 0.000). Therefore, routine disruption favors learning, and entertaining empowers and makes learning easier. A student also said: “*it has been easier learning the concepts and specifications of the different types of training, since it has been a different activity to the ones we are used, we have given more attention and dedication, in the previous preparation as in the observation of other groups in class, at least from my point of view*” (C3).

To summarize, a clear increase (higher than 5.5) in participants' interest in debate and training was observed, with a 10% statistical trust level, although a lower increase was seen in the issue they defended (*p*-value = 0.216). Besides, students considered competitive debate to be very entertaining (*p*-value = 0.000). Although they did not recognize a strong improvement in their communication capabilities (*p*-value = 0.16), the position of students on their improvement in communication skills was above the average. However, if we consult the testimonials written by students, they showed that the interest on the debating issue may be stimulated by the competition effect. Thus, a student said: “*Our group had the opportunity to defend the Corporate Universities. In order to no know very well the issue, all group members felt motivated to look for the maximum possible information and make sure of deepening much, in order to respond any question that we got from the opposing team and being able to make rebuttals*” (C5). Another student offered similar reasoning “*Also, I'd like to remark that the thematic wasn't very appropriate, from my point of view, because it was about learning the concepts of training and, however I just understood mine ones and the ones of the team with which we debated. Simply because we are focusing in debating and winning to other group, more than learning what was each methodology about*” (C6). This allows us to question what would happen if all the debaters dealt with the same issue. A more probable result is that the level of knowledge would increase due to the fact that participants would obtain more information listening to other debates which could be profitable for their own debates and turn into a grading benefit. The competition effect or gamification *per se* promotes putting effort into learning, one student agrees: “*I also believe that the fact of having to prepare this sort of competition, makes you involve more in the research of the issue. I believe that it gets major interest and involvement by some students with practices such as this than the usual presentation in class*“ (C7).

Another student commented in which it was clear that although the debates were an entertaining activity and also demanding, learning also occurred: “*once the two days of debate ended, my conception about this activity changed radically. I had laughed a lot more, it seemed interesting and overall, without realizing, I had very clear differences of each training model. Almost without putting effort into it trying to know what was it about, I had been able to retain a great quantity of information, more than with a regular presentation, sincerely I would have been unable.”* (C8).

Most of the comments mentioned the cost-benefit relation, preparation cost—learning benefit. In this line, another student explained as follows: “*I thought that was a learning way more useful than other methods used in class, since it had a very practical approach and pushed you to learn, not just about the issue that we were debating, but also about the issue of the opposing team. I discovered that this is useful, since generally, if the learning method is something such as presentations, just research the issue that your group must present, but you are motivated to both investigations. I liked the task, since it was a work in a group interaction*” (C9). The findings confirm the positive effect of combining competitive collaboration in gamification, especially when modifying or influencing behavior (Sailer and Homner, 2019). Likewise, the thesis of the same authors is confirmed in which they indicate that collaboration and competition is especially indicated for skills improvement. Therefore, this coincides with Burguillo's thesis in which it is stated that competition can cause social pressure which increases the level of commitment of the participants and can have a constructive effect when participating in learning. In this regard, qualitative testimonials and survey data tell us that having to compete is a catalyst for better performance (Burguillo, 2010). Also, the testimonials of the most motivated students and learning and searching information aligned with the conclusions of Morschheuser et al. (2016) by which inter-team competitions provide clear goals in groups and create clear barriers between groups with positive influences on the group members' individual performances.

Table 2 shows statistical summaries for each one of the analyzed variables.

### Comparison of Debate With Other Grading Possibilities

The usual ways of grading students at this University are memory-based exams, resolution of a practical case, group papers, individual papers, and presentations. The intention of this question consists of measuring whether the debate was better perceived than other grading methods. Thus, students were asked if they thought they learned more or less than with usual grading methods (exams, group papers, individual papers, in-class presentations). The measuring scale ranged from 1 (learned much less) up to 5 (learned much more). The comparison with other grading methods was interesting. Thus, percentages around the possibilities “learned something else” and “I learned much more” accumulated more than half of the responses for exams, papers, and the individual papers with 67, 61, and 51%, respectively. However, compared with the individual presentations, 45% of the respondents said “I have learned something else” and “I have learned a lot more.” These percentage similarities may be due to the fact that classroom presentations are similar to debate. In fact, the response distribution, compared to classroom presentations, accumulated the majority of responses around the option “I learned the same.” Strong results allow us to affirm that competitive debate is better perceived than an exam (*p*-value = 0.002), a group paper (*p*-value = 0.007), an individual paper (*p*-value = 0.000), and even than a classroom presentation (*p*-value = 0.005). Other questions that these results raise include if the learning is real and if objectively measured knowledge acquisition would offer different numbers, especially as the competition effect, in which one is supposed to win, may overshadow learning. Thus, an exchange student declared “*I am a competitive person and activities like this one, are very fun for me. Although is possible that someone may focus too much on winning and not in understanding. The truth is when the opposition was speaking during my debate, all the time I was thinking how to respond for winning and I was not listening to learn. I do not know if this happened to other people, but for me, when is a winner and looser situation I am very short for understanding*.” However, the same student though the activity was more positive than negative in general.

As mentioned, we compared whether the students thought they had learned more about the subject through debate than using other methods (three would mean indifference). The response was clear, students thought that they learned more than doing exams (mean = 3.73, *p*-value = 0.002), papers (mean = 3.79, *p*-value = 0.000), group papers (mean = 3.52, *p*-value = 0.007), or presenting in class (mean = 3.55, *p*-value = 0.005).

All these quantitative data were reaffirmed by qualitative data. Thus, a student expressed “*I am thankful for having such committed professors and, up to the moment, who are aware that the old teaching methods are not good anymore and try to bring into class revolutionary methods that are used in the other side of the world*.” (C11). The debate, as an exam, was perceived as something new and good. The same student observed values and added benefits in debating as a grading method: “*Making students debate and argument about any issue makes them learn more than if the professor relates the contents as if it would be a book. Classroom debate for me is the formalization of the “discussions” generated in class when students ask and question a professor about some issue. The debate generates a critical sense that memorizing a subject does not give you which is important to foment, maybe not so formally (with all of the rules and paraphernalia that debate has, but it does a little approaching)*.” (C12). It is appropriate to notice that this student previously participated in debates.

One student expressed her wariness the activity although acknowledged the fact that presenting before an audience made her look for more information and therefore learn more. However, the general evaluation was positive: “*The debate activity we did, from the beginning scared me a lot. I am a very shy person and public speaking it's hard for me, and to be something so spontaneous and long (5 minutes speaking alone), I was afraid. I was very nervous beforehand, and also right in the moment. But the sensation with which I left after speaking, was of tranquility and complete satisfaction. I would not like to repeat the activity simply because of the nerves, but I admit that is good for us to get out of the comfort zone once for a while (…) at the same time, the fact of being nervous took me to get more information about the issue for being able to defend myself. Therefore, I believe that fulfills the function of learning that the activity had*.” (C13).

A student, reluctant at the beginning, afterwards expressed his appreciation for the activity valuing it better than exams. “*It is a good way to find out about issues that are unknown or little known to us. It foments the search of good arguments, backed up by reliable sources. It is a very dynamic way of learning and touches deeper than a paper or a regular and normal presentation and in which, generally, it's researched about an issue without a purpose apparent. In occasions you learn, but in many other, once the work is finalized, you just start from scratch. However, when debating and confronting arguments you learn much more about an issue or issues that are treated*.” Various initial testimonials relayed reluctancy which then transformed into a positive attitude toward the debating activity: “*Therefore, although at the beginning it shocked me a bit, the issue of debating in HR class, I am glad I did it and see exactly how it was. (…) in conclusion the activity that at the beginning did not seem very interesting since I had never done before something alike, finally, I liked it, enjoyed and learned*.”

The discomfort that comes with participating in a debate is the main reason that provokes learning. Likewise, being a new tool, debate motivates and provokes that exceptional effort. Thus, an exchange student explained: “*Although it was completely different to usual methods, I liked this activity very much. That is because it got me in an uncomfortable position. Being uncomfortable is not the ideal state but is an opportunity for personal growth. Besides, in the future for my career there are going to be cases where possibly I have to do this, then, it is better to start practicing right now. This exercise makes me feel that also allowed me to learn very much. I say this because in order to prepare my debate, I also had to do research about the other group. Then I was learning of the two issues to get ready.”*

### Recommendation of the Activity to Other Students

One of the ways to determine if a training activity has been successful is asking if participants would recommend this activity to other persons of a similar profile. If the activity is recommended the receptiveness has been good and if not, it may be deduced that the experience has been more negative than positive. In this case the majority (88%) would recommend it to similar people. While, 12% would not. In this 12%, a certain bias comes into play as the activity may not be adequate for certain profiles, not because it is not good *per se*. Participants clearly would recommend this activity (proportion = 0.88, *p*-value = 0.000).

The testimonial of a student may explain why he would recommend the activity based on the amusement he found, the engagement that it promoted, and the personal improvement he reached. “*Besides, I found the activity very fun because we could train how to react to questions about an issue that was presented in real time. It was very good that we all took it seriously and had the opportunity to generate tension in the rival group, this made the debates easy to follow. I believe this activity has broken, a bit, the classroom routine and has done well to everyone, because at the same time we explored part of the contents we got in contact with other dynamics such as debate. In the other hand, it has given to shiest people, like me, the opportunity of trying ourselves in front of our mates. Besides, it was very good that we had a previous session so it would be clear the debate format in order to prepare it better*.” (C17). In favor of the debate method explained at the beginning, the student remarked that the previous training was useful.

Another student said that the debate had added value and therefore would recommend the more personal aspect, not only the intellectual or academic value of the activity: “*cognitive and communicative activities were developed and personally, I believe is an activity that prepares students for the moment in which they have to face real situations of debate or possible negotiations. (…) is an activity that brings security and confidence, and this is a fundamental element in people's career, and not only speaking from purely professional point of view, but also personal.”* (C18).

Another element that students were aware of, thanks to qualitative feedback, was if they had to repeat the experience, they would offer different recommendations. Thus, a student explained that, although difficult, the experience had been positive. One of the key points was that the activity was compulsory. “*I am a person to whom results difficult public speaking and this activity has been much more difficult to perform, I did not know how to express myself correctly as I normally do, but for being a first time I believe is normal. If I would have the opportunity to do it again voluntarily, it would be a difficult decision to me and even if I'd think about repeating but because of the simple fact that I do not open myself to public speaking, although probably I would end up doing it because it would help me to improve the way I express myself and the way I trust in myself.”* (C19). Therefore, students who put in a lot of effort also declared an exceptional personal reward in the intellectual and emotional fields.

### Perception of the Debate Activity Validity in a Company for Different Applications

For the future, we intended to discern if actual students, future manager*s*, would consider this training possibility valid or not. Thus, we asked if the activity could be valid in a company for training in communication and making decisions in a team or finding talent, with a dichotomous response (1: Yes; 0: No). Decision-making through debate is applicable from the point of view that a jury has to make a decision about what solution is better out of the two presented. Talent detection it is proposed in order to identify talent when people have to speak in public. This research shows that students would recommend it for training in public speaking with 85% of affirmative responses, followed by talent detection with 73%, and ending with ranking the use for decision-making at 60%.

It is contrasted if the proportion was equal to 0.5 (indifference) and it was observed that students thought that this experience may have validity for a company when teaching communication (proportion = 87.5%, *p*-value = 0.000) and for talent detection (proportion = 75%, *p*-value = 0.007) but there was not a consensus about its validity for decision-making in teams (proportion = 62.5%, *p*-value = 0.217), as can be observed in Tables 3, 4.

**Table 3:** Comparison of the learning sensation with other grading ways and competitive debate.

	**Exams**	**Group papers**	**Individual papers**	**Presentations**
**Scale**	**Percentage**	**Percentage**	**Percentage**	**Percentage**
Much less	9.1	0	3	0
Something less	6.1	9.1	12.1	15.2
The same	18.2	30.3	33.3	39.4
Something more	36.4	33.3	33.3	21,2
A lot more	30.3	27.3	18.2	24.2
Average	3.73	3.52	3.79	3.55
*P*	0.002	0.007	0.000	0.005
Standard deviation	1.23	0.96	1.03	1.03
*N*	33	33	33	33

*Source: self-elaboration*.

**Table 4:** Validity of debate as a training solution for different business skills.

	**Training communication**	**Decision Taking**	**Talent Detection**
**Scale**	**Percentage**	**Percentage**	**Percentage**
Yes	84.8	60.6	72.7
No	12.1	36.4	24.2
**Total**	**100.00**	**100.00**	**100.00**
***N***	32	32	32
***P***	0.000	0.217	0.007

*Source: self-elaboration*.

### Independent Variables

In this part, we checked if the before mentioned variables presented an association or significative difference in relation to the following variables. The following ones are demographic and academic variables: gender, average grade, nationality, enjoys public speaking, won or lost the debate, and previous experience in debate with the idea of finding if there could be any differences in the dependent variable according to the set of independent variables.

Students were asked if they participated in debate activities before this experience. The aim of this question was to discern if the group had any previous experience in competitive debate and if the activity could have had a significative impact. Most of students (85%) had not participated in debate activities previously. There were no variations in any analyzed aspects among students that had performed in a debate and the ones who did not. In fact, there were no differences between teams that won or lost. Out of the five persons that had previously participated in debates there were no differences in whether they won or lost the debate. Thus, three of them won, one lost, and another did not respond about the result.

On the other hand, 51.5% of participants were men and the rest were women, the average grade of participants was 6.96 in a 0–10 grading scale (D.T. = 0.850), and 84.8% of them were of Spanish nationality. There were differences in the average grade (*p*-value = 0.0013), the female students had higher grades (average = 7.44) than the male students (average = 6.63).

There were no differences in the analyzed variables according to gender, except when asked about the result in the debates in which their team participated (*p*-value = 0.035). Thus, among the men, 64.7% of their teams won, and among the women, 25%.

There were no differences in any of the analyzed variables among Spaniards and exchange students, except whether they had learned more with this activity than with exams (*p*-value = 0.045); exchange students disagreed with such an affirmation (mean = 2.80) more than the Spaniards (average = 3.89), and the average grade (*p*-value = 0.074) was higher for the exchange students (average = 9) that the Spaniards (average = 6.88). One explanation might be the use of oral skills in a foreign language and under the pressure of a competition at a certain disadvantage compared to the rest of the majority of the group could have an opposite effect and be perceived as extra difficult.

There was no difference in the analyzed variables between students whose team won or not, except what was mentioned about gender.

No association was detected among proclivity for public speaking with any of the dependent variables. Students from this University were used to doing presentations and the majority of the answers confirmed that the students had no problems when public speaking. Because of this, competitive debate was particularly welcome.

Aside from this, it seemed that a clear positioning in respect to proclivity to public speaking did not exist, this variable was measured according to a 1–4 scale, 1 being “I very much like public speaking”, and 4: “I avoid it if possible.” Since it was not possible to reject the average value of the different values which was 2.5 (*p*-value = 0.808), an average of 2.45 with a standard deviation of 1.063 was apparent. Thus, 24.2% of participants confirmed that they very much like public speaking, another 24.2% said that they can enjoy it, 33.3% declared that they do it if they must, and 18.2% said they would avoid it if possible. These results are in line with Chapman and Rich (2018) who concluded that “Correlational data indicated that being a member of any measured demographic (e.g., gender, age, student status) was not a barrier to finding gamification motivating,” which may explain the lack of potential explanations for this part.

## Conclusions

In general, students that participated in the inter-team gamified competitive classroom debate valued the activity and found it valid for their knowledge and skills development. The majority of students considered this activity to be an adequate method to grade and even better than traditional grading formulas. Likewise, inter-team debate was also seen as an adequate technique for its use in the business environment.

The technique of competitive debate as a gamified activity in the classroom provoked a motivation increase in the students to attend class more than before the experience. The classroom debate, as a new and playful activity, promoted motivational behaviors.

Competitive inter-team gamification through classroom debate increased the interest of students in all of the knowledge areas. They declared an increment of interest for HR training, for the training solution students defended, as well as debating or communication in general. The aspects in which students showed higher interest were communicating and debating. Thus, it may be concluded that, in this experience, the learning perception about debating was higher than the issue of the debate.

Most of the students thought their capacity for public speaking improved after participating in the debates. The gamified inter-team debate technique in the classroom was confirmed as a powerful transversal training method in communication skills. Thus, most of the students, stated that they felt they had improved their public speaking level.

Most of the classroom considered the activity of competitive debate an entertaining learning exercise. To this respect, it may be noted that participants considered it entertaining, but only if they had an active position (judge or debater) and not that much when they were mere spectators.

The debate, as a grading model, was also better received than the traditional formulas such as exams, group papers, and individual papers, although with a similar degree of appreciation in respect to oral presentations in the classroom.

Most of the students would recommend this activity to other students with a similar profile to theirs. This shows the excellent reception of a gamified activity in the classroom such as competitive debate.

Students that joined the experience thought that this technique could be extrapolated to the training business world to teach communication skills or detect talent, and to a lesser extent, although also positively, for decision-making as a team.

Facing implementation and improvement, the training period before the debates was appropriate since it was positively valued in the qualitative part by various students who said that feeling positive helped during the process.

In summary, low technology inter-team competitive gamification offers positive results to motivate and engage students in the learning of HR and the practice of communication.

## Limitations and Future Research

One of the research limitations was the sample size of the survey which was ultimately formed of 33 people, although 42 students participated in the experience. This initial limitation was compensated with the qualitative analysis, which allowed us to tune conclusions. An increase in the sample size is necessary for future research, it could be especially interesting to reiterate the experience, not only with students but with professionals to analyze the impact and receptivity of such a training technique in a professional environment.

For future research, it is possible to study the impact of inter-team gamification as a tool aimed at effectiveness in terms of knowledge acquisition, thus, an investigation can be carried out in which the acquisition of knowledge is measured with a multiple choice exam and with a control group. It is possible to carry out experiments for the acquisition of skills and knowledge in the business world.

Another possible investigation would consist of expanding the assessment of common elements in gamification such as points, badges, or leaderboards during the debate process which probably would require high technology. Likewise, in this line, it would be necessary to investigate the perception of the students with respect to the levels of difficulty through inter-team gamified debate.

Another possible research focus would be a bibliometric analysis and of the current state of inter-team gamification due to the scarcity of research in this regard.

The use of inter-team gamification, through debate or other mechanics, in order to assign grades remains unresearched.

## Data Availability Statement

The raw data supporting the conclusions of this article will be made available by the authors, without undue reservation.

## Ethics Statement

Ethical review and approval was not required for the study on human participants in accordance with the local legislation and institutional requirements. Written informed consent for participation was not required for this study in accordance with the national legislation and the institutional requirements.

## Author Contributions

GS, MM, and AR conceived the research, designed the article, and developed all the tables and figures used in this article. All authors contributed to the article and approved the submitted version.

## Conflict of Interest

The authors declare that the research was conducted in the absence of any commercial or financial relationships that could be construed as a potential conflict of interest.

## Publisher's Note

All claims expressed in this article are solely those of the authors and do not necessarily represent those of their affiliated organizations, or those of the publisher, the editors and the reviewers. Any product that may be evaluated in this article, or claim that may be made by its manufacturer, is not guaranteed or endorsed by the publisher.
